# Movement Complexity and Neuromechanical Factors Affect the Entropic Half-Life of Myoelectric Signals

**DOI:** 10.3389/fphys.2017.00679

**Published:** 2017-09-19

**Authors:** Emma F. Hodson-Tole, James M. Wakeling

**Affiliations:** ^1^School of Healthcare Science, Manchester Metropolitan University Manchester, United Kingdom; ^2^Department of Biomedical Physiology and Kinesiology, Simon Fraser University Burnaby, BC, Canada

**Keywords:** EMG, sample entropy, locomotion, motor control, cycling

## Abstract

Appropriate neuromuscular functioning is essential for survival and features underpinning motor control are present in myoelectric signals recorded from skeletal muscles. One approach to quantify control processes related to function is to assess signal variability using measures such as Sample Entropy. Here we developed a theoretical framework to simulate the effect of variability in burst duration, activation duty cycle, and intensity on the Entropic Half-Life (EnHL) in myoelectric signals. EnHLs were predicted to be <40 ms, and to vary with fluctuations in myoelectric signal amplitude and activation duty cycle. Comparison with myoelectic data from rats walking and running at a range of speeds and inclines confirmed the range of EnHLs, however, the direction of EnHL change in response to altered locomotor demand was not correctly predicted. The discrepancy reflected different associations between the ratio of the standard deviation and mean signal intensity ($Is_{t}:\bar{I_{t}}$) and duty factor in simulated and physiological data, likely reflecting additional information in the signals from the physiological data (e.g., quiescent phase content; variation in action potential shapes). EnHL could have significant value as a novel marker of neuromuscular responses to alterations in perceived locomotor task complexity and intensity.

## Introduction

Good locomotor function is essential for survival in the majority of animals and depends on appropriate functioning of the neuromusculoskeletal system. The majority of scientific work investigating neuromuscular function has however focused on studying steady-state, isometric contractions, and as such underlying principles that govern appropriate neuromuscular responses to tasks of different complexity and intensity are difficult to establish.

In skeletal muscles, the motor unit is defined as an α-motor neuron, its axon and all the muscle fibers it innervates (Sherrington, 1925). Motor unit activity can be detected from changes in muscle fiber membrane potential, triggered by the arrival of impulses from the central nervous system, and recorded using electromyography. The resulting myoelectric signal is the interference pattern produced by superposition of motor unit action potentials within the detection volume of a recording electrode, and can be considered to represent the command signals sent from the central nervous system.

Terrestrial gait patterns are cyclical in nature, with the limbs following a repeated series of movements to support and move the animal's body weight against gravity. The behavior of the majority of limb muscles during any given gait is therefore often characterized by alternate periods of activity and quiescence, occurring over distinct periods of the limb kinematics. The timing, duration, and amplitude of activity are influenced by the mechanical demands of the gait (e.g., velocity) and external environmental factors (e.g., substrate and terrain). In addition, mechanical demands of the movement can influence the number and population of motor units recruited during the activation phase (Wakeling, 2009b). For any given movement cycle, there must therefore be co-ordination of: (i) the relative timing and duration of activation and quiescence (myoelectric duty cycle) and (ii) recruitment and firing characteristics of activated motor units within muscles and across muscle groups. Such features of neuromuscular co-ordination should be represented within recorded myoelectric signals. The complexity of signals recorded during tasks such as locomotion, however make useful quantification of fluctuations in such characteristics challenging.

Assessment of the features of variability within a signal can be used to quantify the underlying control process (Harris and Wolpert, 1998). Entropy provides a practical method of quantifying the complexity of a system and/or signals produced by it (Pincus, 1991; Richman and Moorman, 2000). One entropy based measure, which is suitable for application to non-stationary physiological time series such as myoelectric signals, is sample entropy (SampEn) (Richman and Moorman, 2000). Smaller SampEn values are considered to represent more order and greater values indicate more randomness within recorded signals. This approach has recently been applied to myoelectric data to provide indicators of post-stroke spasticity (Yeh et al., 2016) and motor unit remodeling due to aging (Siddiqi et al., 2015). Recently Zandiyeh and Von Tscharner (2013) introduced a novel method of identifying the transition between SampEn values reflecting order to those reflecting randomness within a signal, termed Entropic Half Life (EnHL). The reshape scale method they proposed reorganizes the time series over multiple scales (number of data points) to determine the time scale over which subsequent data points remain affiliated to one another. As such this approach provides a means of quantifying short term fluctuations in a signal that can describe adjustments in the underlying signal process, and are applicable to studying neuromuscular function during motor tasks.

Quantifying EnHL in myoelectric signals, recorded across multiple muscles during cycling exercise in humans, has revealed increased persistence in signal structure in response to increased cycling load (Enders et al., 2015). These results were suggested to indicate more structured and orderly motor unit firing patterns occurred at higher effort levels (Enders et al., 2015). To our knowledge however, this is currently the only study to quantify EnHL in relation to neuromuscular co-ordination. No previous work has evaluated the features of myoelectric signals that may influence changes in the EnHL metric. To understand the potential value and limitations of this novel approach for investigating neuromuscular responses to task demand, a structured investigation of the features of myoelectric data that influence EnHL values is required and forms the basis of the work presented here.

### Summary of proposed approach

The overarching aims of this study were to: (i) assess myoelectric signal features that influence measures of persistence in signal structure; and (ii) evaluate the resulting predictions of structure persistence using previously reported physiological data. The outcome measure of persistence in signal structure chosen to be evaluated was EnHL, calculated using the reshape scale method and sample entropy based analysis proposed by Zandiyeh and Von Tscharner (2013) and Enders et al. (2015). To achieve these aims the methods were split into two sections. The first involved theoretical analysis of factors that influence EnHL, using synthetic signals generated using an analytical modeling approach. In the second section results from the theoretical work were used to predict EnHLs that may occur across a range of physiologically realistic muscle activation characteristics. This was achieved by collating activation characteristics (i.e., activation duration and duty cycle) previously reported in the literature for cycling and running based locomotor tasks. We then evaluated the predicted EnHLs by comparing them to EnHLs gained from reanalysis of myoelectric data recorded from ankle extensor muscles of treadmill running rats. The following specific objectives were therefore set:

- Generation of a series of synthetic myoelectric signals with known motor unit activation and firing statistics across a range of activation levels, durations and duty cycles (defined as ratio of activation duration to movement cycle duration);
- Calculation of EnHLs for each generated signal and identification of activation parameters that influenced values;
- Identification of the space of modeled EnHLs that occur across the sampled activation duration and duty cycles;
- Prediction of EnHLs for different locomotor activities, based on activation durations and duty cycles reported in the literature for human cycling (Blake and Wakeling, 2015) and running rats (Hodson-Tole and Wakeling, 2008a, 2010);
- Comparison of predicted EnHL to those obtained from new analysis of myoelectric data, recorded from ankle extensor muscles of rats during treadmill locomotion at a range of velocity and incline conditions.

## Methods

### Theoretical analysis of factors influencing EnHL of myoelectric signals

The following sections detail the approaches used to: (i) generate simulated myoelectric signals with defined activation levels, durations and duty cycles; and (ii) analyse these simulated signals to predict EnHL across physiologically realistic activation patterns.

#### Skeletal muscle model properties

Following the protocol used by Wakeling (2009a), an abstracted model representing a simplified human medial gastrocnemius muscle was established. It had a cylindrical muscle belly of radius 17.9 mm and length 150.0 mm and distal and proximal tendons of length 50.0 and 30.0 mm (with 2.0 mm standard deviation), respectively. Skin and fat layers (2.0 and 1.0 mm thick, respectively) surrounded the muscle, while the motor endplates were positioned 20.0 mm proximal to the mid-point of the muscle (with 1.0 mm standard deviation).

Three hundred motor units, each with a unique activation threshold (between 0 and 0.8), were randomly positioned within the muscle belly up to a depth of 20.9 mm below the recording point on the skin. Six models with randomly generated motor unit distributions within the muscle were generated. The MG in humans has been reported to contain ~50% slow and 50% fast fibers (Johnson et al., 1973). Motor unit action potential conduction velocities were therefore modeled using a Gaussian distribution of 4.5 ± 1.1 m s^−1^, resulting in the bottom 50% of motor units having a mean conduction velocity of 3.6 m s^−1^ and the top 50% having a mean conduction velocity of 5.4 m s^−1^, matching predictions of motor unit action potential velocities from previous work (Sadoyama et al., 1988).

#### Firing statistics

Motor unit firing statistics were governed by a neural excitatory (*ne*) drive with recruitment and de-recruitment patterns following an orderly sequence in accordance with Henneman's size principle theory of motor unit recruitment (Henneman et al., 1965). Firing statistics for repeated cycles of activity and quiescence over a 15 s period were created. Activation level, defined as the number of activated motor units, was set to between 0.2 and 1.0 (0.2 increments) of the 300 units included in the model. Activity burst durations between 150 and 900 ms (with 150 ms increments) and duty cycles between 30 and 90% total cycle duration (with 20% increments) as well as 100% total cycle duration were created. These conditions replicate reported values across a wide range of locomotor activities, including cycling humans (Blake and Wakeling, 2015) and running rats (Hodson-Tole and Wakeling, 2008a, 2010).

During quiescence *ne* was zero. Activity bursts were created using ramp increases/decreases in *ne* to/from the required activation level (0.2–1.0), with the activation and deactivation phases each accounting for 50% of activity burst duration. Each motor unit (activated for the given activation level) was excited when *ne* exceeded its threshold. Initial firing frequency (i.e., when the unit was just excited) was 5 Hz increasing to a maximum value of between 25 and 30 Hz when *ne* was 0.2 greater than the threshold, ensuring that all units fired at a maximum frequency when *ne* was 1. Higher threshold units were assigned greater firing rates.

To determine the influence of variation in motor unit firing patterns two sets of firing statistics were created. In one, each firing point was given white noise variation resulting in mean standard deviation firing rates of 14.20 and 85.85 Hz for first and last recruited units in 100% activation state, respectively. While these standard deviations are larger than typically reported in the literature, a second set of firing statistics even larger amplitude white noise was added, leading to signals with even greater variability in the firing pattern, with mean standard deviation firing rates of 22.59 and 124.41 Hz for first and last recruited units in 100% activation state, respectively. Two sets of five firing statistic records were devised for each condition, providing a series of activation-deactivation cycles with a range of activity durations and duty cycles at five different levels of activity, that were next combined with a volume conductor model to simulate surface myoelectric signals.

#### Surface EMG simulation

The potential distribution of each motor unit action potential at the skin was simulated using a freely available analytical three-layer volume conductor model (Blok et al., 2002). Conductance through the anisotropic muscle tissue was 0.1 and 0.5 S/m for radial and axial conductivity, respectively, 0.05 S/m through the fat layer and 0.75 S/m through the skin. The simulated bioelectric source was an intracellular action potential calculated (Rosenfalck, 1969) for a muscle fiber aligned parallel to the axis of the cylinder. Each motor unit contained 300 muscle fibers and the motor unit action potential output from the volume conductor model was sampled at 2,000 Hz. Surface myoelectric interference patterns, calculated for bipolar surface electrodes (10 mm diameter, 22 mm inter-electrode distance), were created by using the firing statistic records to determine addition of motor unit action potentials at appropriate points in time. Signals were determined for a total of 9,000 trials (2 firing statistic variations × 6 muscle models × 6 activation burst durations × 5 duty cycles × 5 activation levels × 5 repeats).

#### Analysis of simulated signals

##### Wavelet analysis and filtering

A filter bank of 11 non-linearly scaled wavelets (indexed by 0 ≤ *k* ≤ 10) was used to decompose signals into their intensities, *I*, as a function of time and frequency. Each wavelet (φ(*f*)) was defined using the function

(1) $$\begin{array}{l} \varphi \left(f\right)={\left(\frac{f}{f_{c}}\right)}^{f_{c}\cdot scale}e{\left(\frac{-f}{f_{c}}+1\right)}^{\cdot f_{c}\cdot scale} \end{array}$$

where *f*_*c*_ represents the center frequency and *scale* is a scaling factor defining the width/shape of the function (Von Tscharner, 2000), here defined as 0.3. At each time point the intensity of the signal at each wavelet domain was calculated from the magnitude and the first time-derivative of the square of the convolution of the raw signal and the wavelet. To align with steps taken to assess experimental data, where additional sources add non-physiological low frequency noise to the signal (e.g., electrode skin interface), wavelet *k* = 0 was discarded from further analysis so data taken forward spanned a frequency bandwidth of ~11–432 Hz. The total intensity (*I*_*t*_) at a given time was calculated by summing the intensities over the included wavelets and can be considered a close approximation to the power of the signal within a given frequency band, comparable with twice the square of the root mean square (amplitude) value (Wakeling et al., 2002). For each signal the mean ($\bar{I_{t}}$) and standard deviation (*I*_*t*_*s*) of the total intensity and the ratio $I_{t}s:\bar{I_{t}}$ were calculated.

As part of SampEn based analyses it is important to determine whether the same results would be obtained if an independent, but identically distributed random variable were analyzed. To do this phase randomized surrogate data were created from the original simulated signals. Each simulated signal was Fourier transformed before the phase was randomized and an inverse Fourier transform applied to generate the time series surrogate signal. This process ensured the surrogate signals did not contain structure related to signal phase, but did retain the same power spectrum and auto-correlation as the original simulated signals (Enders et al., 2015). To this end the mean frequency of the original and phase randomized signals were also calculated:

(2) $$\begin{array}{l} f_{m}=\frac{\sum_{k}f_{c}\left(k\right)I_{k}}{\sum_{k}I_{k}} \end{array}$$

##### Sample entropy and entropic half-life calculations

SampEn(*m, r, N*) quantifies the regularity of a time series of length *N*. It reflects the conditional probability that two sequences of *m* consecutive data points, that are similar to each other within a tolerance of *r*, will remain similar when one more, consecutive, data point is added (Richman et al., 2004). This can be considered to be a match count process that is repeated across the analyzed time series:

(3) $$\begin{array}{l} SampEn=\;-log\left(\frac{\left(\sum A_{i}\right)}{\left(\sum B_{i}\right)}\right)=-log\frac{A}{B} \end{array}$$

where *A*_*i*_ represents the count of matches with the *i*^*th*^ template of length *m* + 1 and *B*_*i*_ represents the count of matches with the *i*^*th*^ template of length *m* to provide a unitless, non-negative number. SampEn values approaching zero reflect highly periodic time series, while greater values indicate more variable signal waveforms. To minimize the effect of the regular activation bursts on SampEn calculations, the total intensity envelope of each signal was down sampled to 1,000 Hz and filtered using a 10 Hz highpass Butterworth filter.

**Figure 1:**
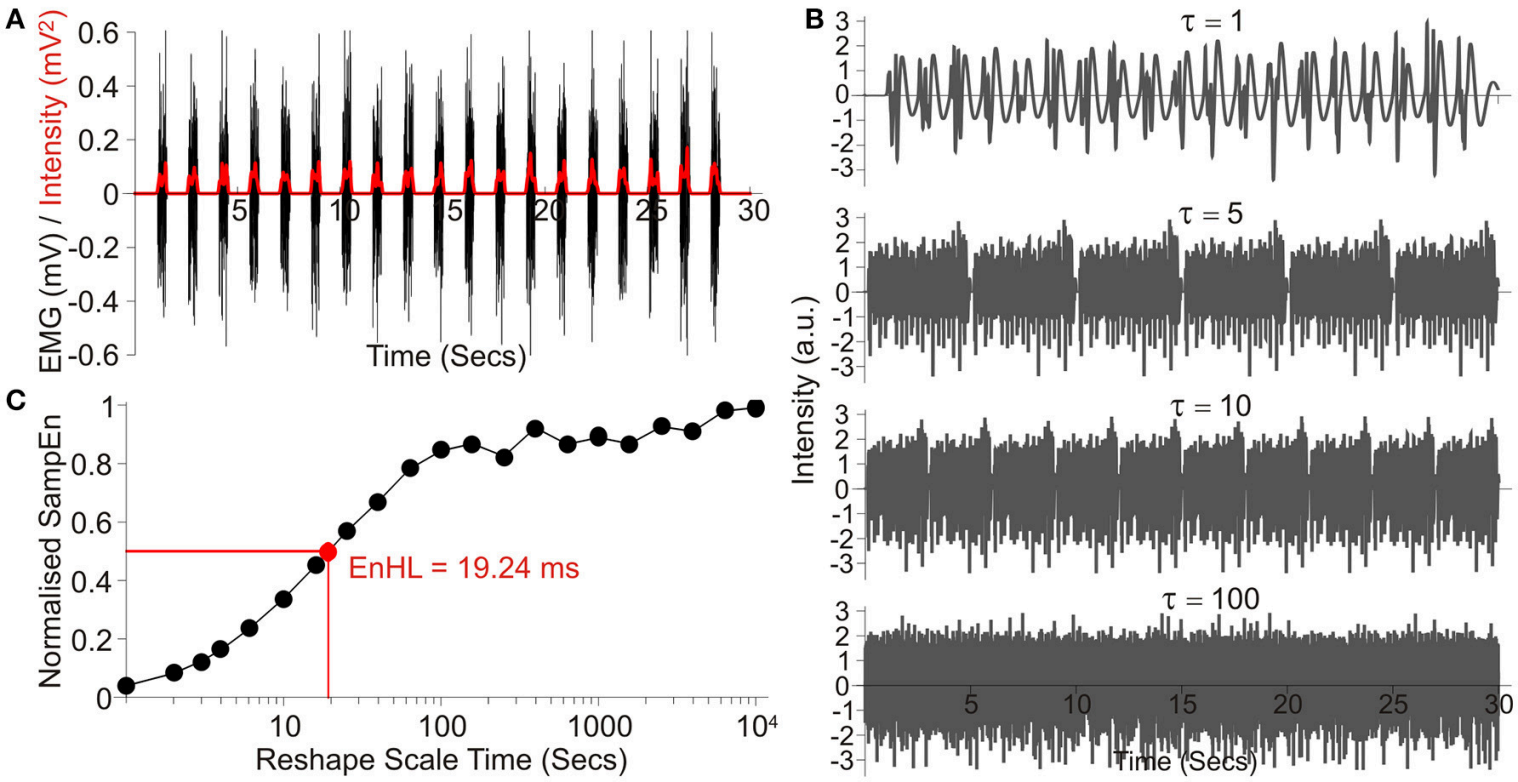
Overview of processing steps, using an example signal taken from those simulated showing: **(A)** the raw signal (Black) and the intensity envelope (Red, *I*_*t*_) resulting from its wavelet transformation; **(B)** a series of four signals created by resampling the standardized and filtered intensity envelope at increasing time steps, denoted by τ; **(C)** SampEn values resulting from analysis across the entire τ range, normalized to SampEn from the signal after random permutation of all data points. The resulting EnHL (Normalized SampEn = 0.5) is highlighted (Red point).

A freely available software package (Goldberger et al., 2000) was used to calculate SampEn for the filtered total intensity envelope of each signal. Prior to the SampEn calculation a reshape scale method was used to reshape the time series to provide increasingly larger time intervals (Δ*t*) between consecutive data points, determined by a τ -scale (Zandiyeh and Von Tscharner, 2013; Enders et al., 2015),

(4) $$\begin{array}{lll} P_{i} & = & \left[x_{i+0\times \tau },x_{i+1\times \tau },\;x_{i+2\times \tau },\;x_{i+3\times \tau },\;x_{i+j\times \tau }\;\right],\\ where\;\left\{\forall j\;\in {\mathbb{Z}}^{\geq 0}|\;j\times \tau +i\leq L\right\},i=1,\;2,\;\ldots \;,\tau \end{array}$$

where *x*_*i*_ is the *i*^*th*^ data point of the original signal (Zandiyeh and Von Tscharner, 2013). As described by Enders et al. (2015) the signal at scale τ was constructed by randomizing and appending the *P*_*i*_ s, with randomization of the blocks preventing reordering of elements at larger scales (Figure 1).

Each signal was standardized to have a mean of zero and standard deviation of 1. The signal was then rescaled using τ between 1 and 10,000 data points, representing timescales of 1 ms to 10 s and the SampEn for each case calculated (*m* = 1, *r* = 0.2). The values of *m* and *r* were selected to ensure the whole range of low and high SampEn values were observed across the τ-scale (Figure 1; Zandiyeh and Von Tscharner, 2013), whilst maximizing the deviation from *m* = 0 (equivalent to data being completely randomized) when τ = 1 (original time series order; Richman et al., 2004). Resulting values were normalized to the maximum SampEn calculated for the signal after random permutation of all data points was completed prior to filtering and SampEn calculation (equivalent to calculating SampEn with *m* = 0).

Normalized SampEn values were plotted as a function of timescale τ and the timescale at which the curve crossed half its maximum value was identified as the EnHL (Figure 1; Zandiyeh and Von Tscharner, 2013). This value represents the time scale at which the transition from short time interval, non-random, orderly amplitude differences to larger time interval, random amplitude differences occurs. Finally, the resulting EnHLs were plotted as a function of activation duration and duty cycle, providing a map of values from which EnHLs could be predicted for physiological data with known activation durations and duty cycles. All data analysis was completed using custom written code (Wolfram Mathematica 9, Wolfram Research Inc.).

#### Statistical analysis of EnHL in simulated signals

The null hypothesis of no significant difference in the phase (structure) of original and phase randomized signals was tested using a Wilcoxon signed-rank test to compare EnHLs from the two groups. This approach was selected as Kolomogorov–Smirnov test of normality revealed EnHLs were not normally distributed within the original or phase randomized simulated signals (*p* < 0.001, both cases). To further investigate differences in signal structure across the parameters manipulated in the simulations, Wilcoxon signed-rank tests were completed on values from each duty cycle/activation duration grouping with the critical value adjusted to α = 0.0017 (0.05/[5 duty cycle × 6 activation durations]). In addition, differences between EnHL values resulting from the two firing statistics were also evaluated using Wilcoxon signed-rank test.

The distribution of data made application of statistical tests to determine effects of simulation parameters challenging. As such, differences in signal properties (mean ($\bar{I_{t}}$) and standard deviation (*I*_*t*_*s*) of signal intensity; ratio of the standard deviation and mean signal intensity ($I_{t}s:\bar{I_{t}}$); SampEn) across simulation parameters and associations between them have been inferred from observation of graphical plots.

### Prediction of EnHL in physiological data

The following section details the steps taken to determine EnHL in physiological myoelectric data recorded from: (i) lower legs of humans during cycling; and (ii) ankle extensor muscles of rats during treadmill running. These analyses were completed to predict EnHLs likely to occur across physiologically realistic ranges of activation patterns. In addition details pertaining to the reanalysis of myoelectric signals recorded from ankle extensor muscles of treadmill running rats, completed to enable direct comparison between simulated and physiological EnHLs, are also provided.

#### Data from human cycling

The only previous report of EnHL quantified in myoelectric signals used data from lower limb muscles of human males, recorded during cycling (90 rpm, 150 and 300 W; Enders et al., 2015). This report focused on deriving EnHL as a feature of multi-muscle co-ordination, rather than within individual signals as is the focus here. Signal characteristics of interest here (i.e., activation duration and duty cycle) were therefore not reported. Blake and Wakeling (2015), however have reported related activation patterns of 10 lower limb muscles of male cyclists, recorded during cycling at cadences of 40–180 rpm (20 rpm intervals) each at 100–400 W (100 W intervals) on an indoor cycle trainer (Blake and Wakeling, 2015). Activation durations and duty cycles from 10 muscles × 4 power × 8 cadence conditions were therefore plotted within the map of EnHLs, resulting from analysis of simulated signals. Specifically a point marking the intersection of each activation duration and duty cycle value recorded by Blake and Wakeling (2015) was plotted onto the space of modeled EnHLs, and the corresponding EnHL value noted for the each muscle × power × cadence of the cycling data.

#### Data from running rats

From previously reported work (Hodson-Tole and Wakeling, 2007, 2008a,b, 2010), we had myoelectric data from soleus (SO), plantaris (PL), and medial gastrocnemius (MG) muscles of 10 rats (*Rattus norvegicus*) recorded during treadmill locomotion at nine velocity and incline combinations (0° at 20, 30, 40, 50 cm s^−1^; 10° at 20, 30, 40 cm s^−1^; 20° and 25° at 20 cm s^−1^). These data were used in two ways. Firstly, as was done with data from human cycling, reported values for myoelectric activation duration and duty cycle (Hodson-Tole and Wakeling, 2008a, 2010) were plotted onto the EnHL map, created from the analysis of simulated signals. This provided predictions of EnHL for the range of locomotor velocity and incline conditions studied. Secondly, recorded myoelectric signals from each of the three muscles were reanalyzed to provide measures of EnHLs, enabling direct comparison of predicted EnHLs between simulated and physiological results.

As described in the previous publications (Hodson-Tole and Wakeling, 2007, 2008a), and in a similar way to the simulated signals here, a filter bank of 20 non-linearly scaled wavelets (indexed by 0 ≤ *k* ≤ 19) was used to decompose experimental myoelectric signals into their intensities. Wavelet domains 0 ≤ *k* ≤ 3 were discarded from further analysis, so that *I*_*t*_ of the signal was calculated across the frequency bandwidth of ~70–1,325 Hz. In addition to *I*_*t*_, the intensity within low (*I*_*L*_) and high (*I*_*H*_) frequency signal components were also quantified. This was achieved by applying two wavelets, defined using Equation (1) (*c*_*f*_ = 206.28 and 457.99; *scale* = 0.045 and 0.034), optimized to quantify frequency components within each region, as described in Hodson-Tole and Wakeling (2007). Each optimized wavelet had a time resolution of 2.50 ms, achieved by multiplying the originally defined ones with a Gaussian filter (μ = 0, σ = 1.2). *I*_*L*_ and *I*_*H*_ were quantified to enable investigation of whether EnHL differs between the distinct myoelectric signal frequency components that can be interpreted as representing activity in slower and faster motor unit populations (Wakeling et al., 2002; Hodson-Tole and Wakeling, 2009).

Total (*I*_*t*_), low frequency (*I*_*L*_) and high frequency (*I*_*H*_) intensities were calculated for each muscle in each experimental condition. The mean, standard deviation, and ratio of the standard deviation and mean signal intensity were calculated for each component. As in the analysis of the simulated signals, intensities were down sampled to 1,000 Hz and filtered with a 10 Hz highpass Butterworth filter to remove the underlying repetitive temporal structures caused by the stride frequency. Filtered signals were analyzed as described in Section Sample Entropy and Entropic Half-life Calculations, providing EnHL for each. To ensure results of this analysis reflected structure within the physiological signals, EnHL values were also determined for phase randomized surrogate signals, with one signal produced for each trial/muscle/subject combination.

##### Statistical analysis of EnHL from rat running data

Kolmogorov–Smirnov tests of normality revealed that the median EnHL values recorded from all subjects were not normally distributed. Normality was however achieved through Log transformation of values. Mixed effects linear models were therefore fit to the Log transformed values (IBM SPSS Statistics 22). This approach enabled assessment of the influence of each fixed factor, without inclusion of errors that would result from the likely correlations between within-subject values (i.e., repeated measures from subjects across conditions). Analyses were initially run across the whole data set and then run separately for each intensity component. The process involved full factorial inclusion of all factors (Fixed: muscle, incline, velocity, intensity component [analysis of pooled results]) with separate intercepts modeled for each parameter and subject (defined as the random factor). While statistical tests were run on the Log transformed data, values reported are the median ± interquartile range of original EnHLs.

## Results

### Theoretical evaluation of factors influencing EnHL

**Figure 2:**
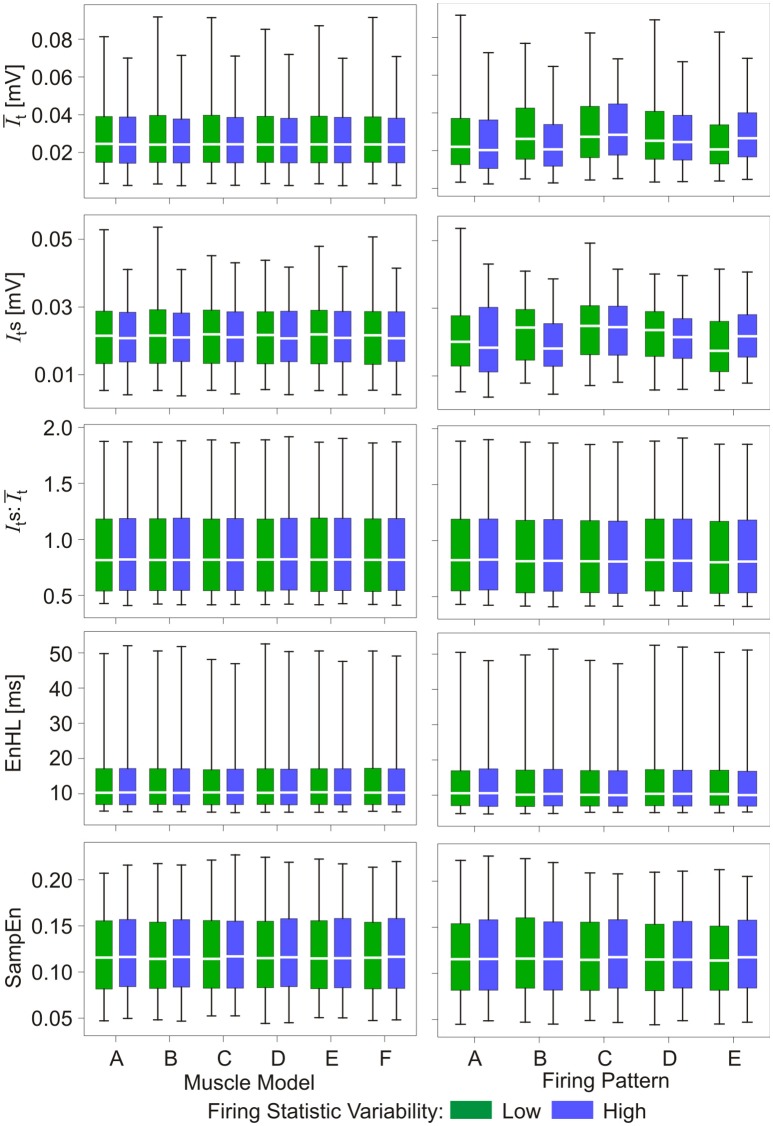
Box and whisker plots of characteristics of simulated myoelectric signals, with each row representing data for a different characteristic. Values are grouped according to six muscle model repeats (A–F, left column) and five firing pattern repeats (A–E, right column). Green bars represent data from the low variability firing patterns; blue bars represent data from the greater variability firing patterns. Mid-box marker represents the group median. Upper and lower box edges represent 75 and 25% quantiles, respectively. Upper and lower whiskers represent maximum and minimum values, respectively.

**Figure 3:**
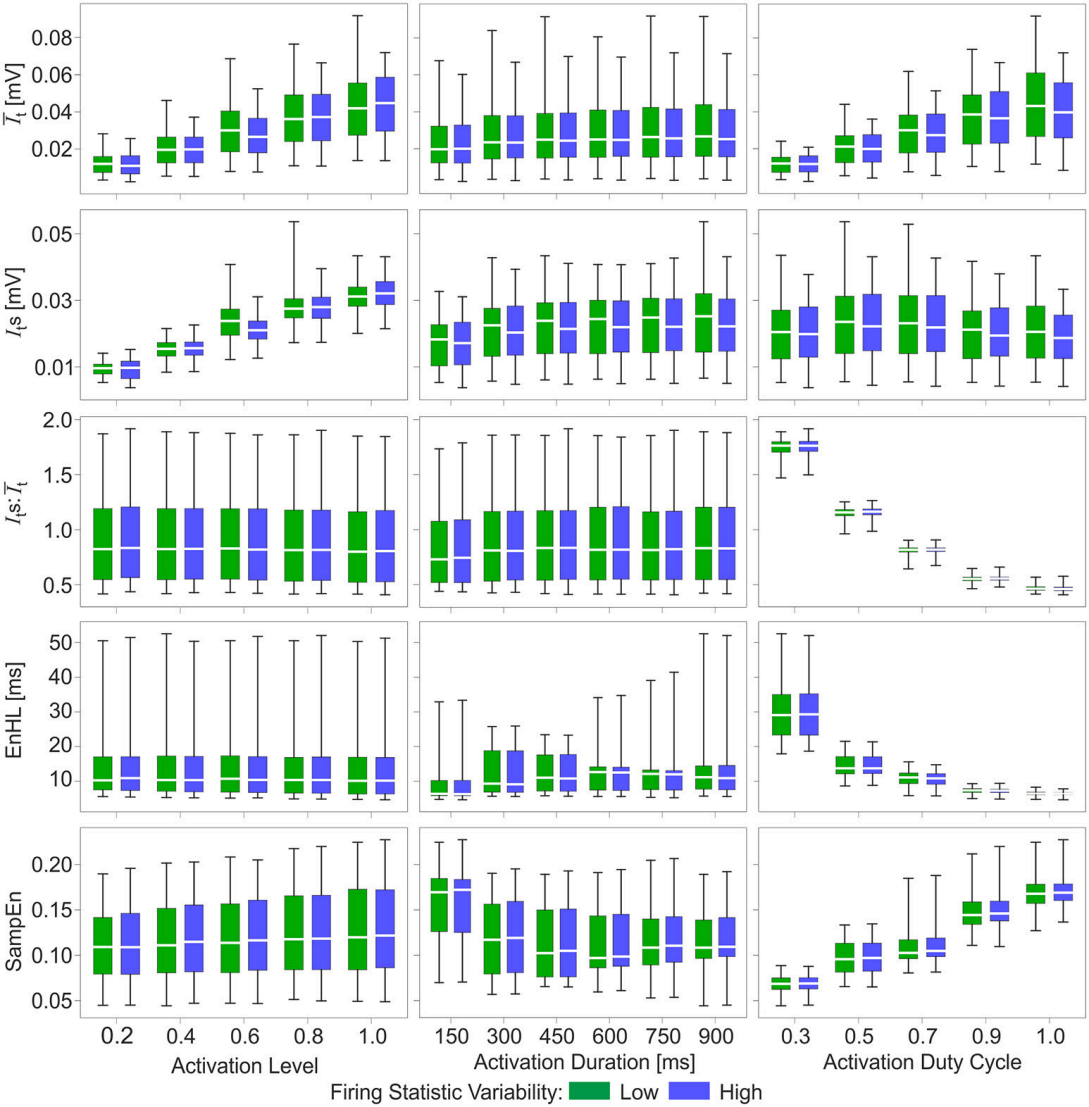
Box and whisker plots of characteristics of simulated myoelectric signals, with each row representing data for a different characteristic. Columns represent the activation features manipulated to generate the signals: activation level **(left)**, activation duration **(middle)**, and activation duty cycle **(right)**. Green bars represent data from the low variability firing patterns; blue bars represent data from the greater variability firing patterns. Mid-box marker represents the group median. Upper and lower box edges represent 75 and 25% quantiles, respectively. Upper and lower whiskers represent maximum and minimum values, respectively.

Results from the analysis of simulated myoelectric signals are presented in Figures 2, 3 (Also see Supplementary Material). Figure 2 shows there were no distinct differences between the properties of signals simulated using the different muscle model repeats nor between the different firing pattern simulations. In addition, differences in the variability within the firing statistics had no effect on measured signal features.

**Figure 4:**
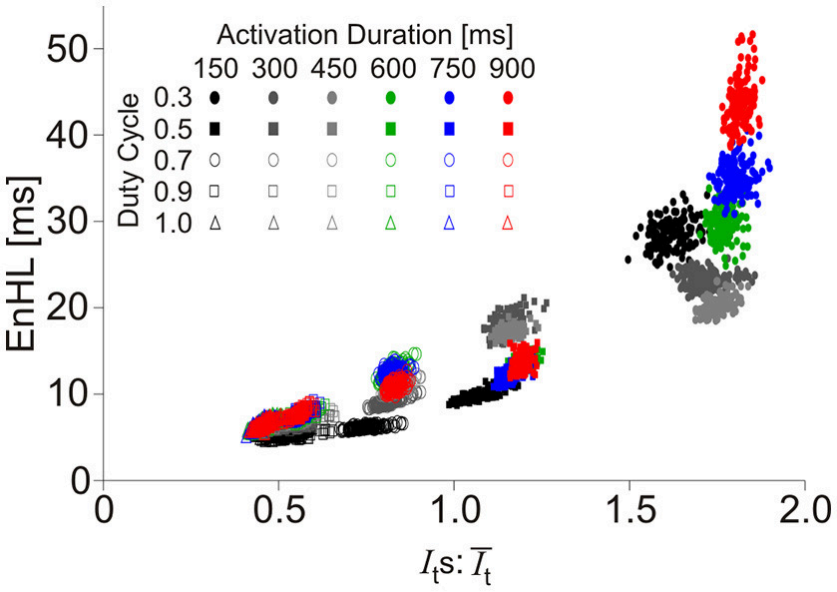
Scatter plot of EnHL as a function of $I_{t}s:\bar{I_{t}}$ for each of the activation duration and duty cycle parameters manipulated in the creation of synthetic myoelectric signals.

Of the activation characteristics manipulated across the simulations (Figure 3), activation level had an effect on the mean ($\bar{I_{t}}$) and standard deviation (*I*_*t*_*s*) of the signal intensity, with increasing activation associated with higher $\bar{I_{t}}$ and *I*_*t*_*s*. The ratios of $I_{t}s:\bar{I_{t}}$ were similar across activation levels. There was no effect of activation level on EnHL or SampEn. Activation duration did not influence $\bar{I_{t}}$ or *I*_*t*_*s* and there were no differences in SampEn across durations studied. In contrast, activation duty cycle had a clear influence on EnHL, with longer EnHL occurring at lower duty cycles. Larger $I_{t}s:\bar{I_{t}}$ values were also seen at lower duty cycles, significantly decreasing as duty cycle was increased. In contrast, SampEn values increased from the lowest to the highest duty cycles assessed. The relationship between EnHL and $I_{t}s\;:\;\bar{I_{t}}$ is shown in Figure 4 and shows that EnHL is predicted to increase with $I_{t}s:\;\bar{I_{t}}$. The longest EnHL therefore resulted from a combination of the longest activation durations and shortest duty cycle values assessed.

EnHL from the original signals were significantly larger than from the phase randomized signals (*p* < 0.01). Assessment of EnHL within signal duty cycle and activation duration groupings revealed significant differences occurred in all groups (*p* ≤ 0.0017), except where duty cycle was 1 and activation duration 300 or 450 ms (*p* = 0.70 and *p* = 0.02, both cases). There was a strong, negative relationship between the mean frequency and EnHL of the phase randomized signal (*r*^2^ = 0.74), which did not exist in the original signal (*r*^2^ = 0.0001) despite there being no difference in the mean frequency of the original and phase randomized signals (*p* = 0.09).

**Figure 5:**
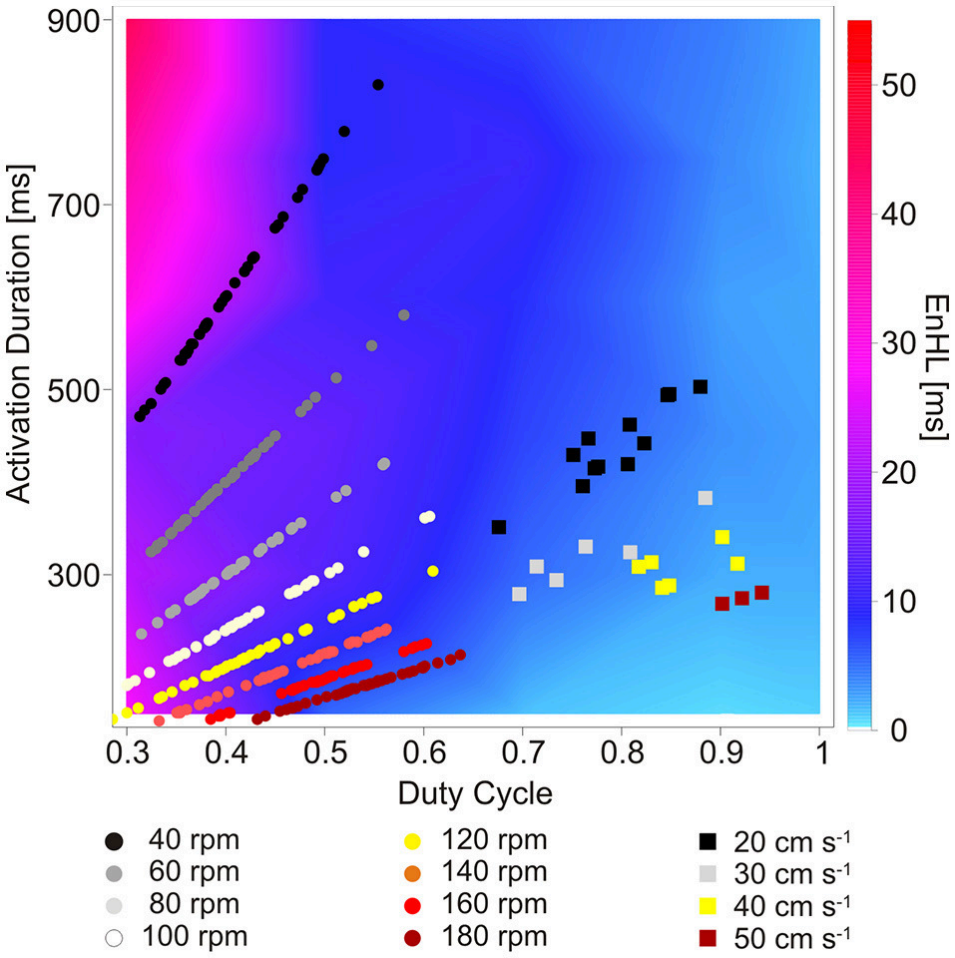
Map of EnHLs as a function of activation duration and duty cycle. Warmer colors represent longer EnHLs. Overlaid data points represent activation durations and duty cycles reported for lower limb muscles of humans cycling (Blake and Wakeling, 2015) (circles) and rats running on a motorized treadmill (Hodson-Tole and Wakeling, 2008a, 2010) (squares). Black-gray tones represent faster cadence/velocity; yellow-red tones represent slower cadence/velocity.

The map of EnHLs, predicted to occur across the activation durations and duty cycles studied, is shown in Figure 5. These data were taken from the 100% activation case, simulated with the greatest variability firing statistics (plots were extremely similar across all activation/firing statistic levels studied, as indicated by grouped results in Figures 2, 3). Predicted EnHLs ranged between 4.66 and 52.01 ms. As would be expected from Figure 4, the largest EnHL (>45 ms) are predicted to occur for activation patterns with the longest duration (900 ms) shortest duty cycle (0.3) values. The shortest EnHLs (<5 ms) are predicted to occur for conditions associated with longer duty cycles (>0.8).

### EnHLs predicted to occur in physiologically relevant conditions

Overlaid onto the predicted EnHL activation duration/duty cycle map (Figure 5) are data points reflecting activation durations and duty cycles reported to occur in human lower limb muscles during cycling (round symbols, Blake and Wakeling, 2015) and ankle extensor muscles of rats running on a treadmill (square symbols, Hodson-Tole and Wakeling, 2008a, 2010). Points from the cycling data (Blake and Wakeling, 2015) provided a large range of activation durations coupled with duty cycle values below 0.65, which resulted in all data points lying to the left of the mapped parameter space, predominantly in the lower left quadrant (Figure 5). Myoelectric signal characteristics were clearly differentiated by cycling cadence, with the slowest cadences associated with the longest activation durations. This separation across data points resulted in a much larger range of EnHL values being predicted than was seen for the rodent running data. The longest predicted EnHLs (40.00 ms) were associated with the intermediate cadence conditions (100, 120 rpm) where short activation durations and duty cycles occurred. The shortest EnHLs (9.94 ms) were associated with the fastest cycling cadences (180 rpm).

Data points from the treadmill running rats occupy the lower right quadrant of the mapped parameter space, reflecting high duty cycle (>0.5) and moderate activation durations (275–505 ms). The location of these data points within the activation-duty cycle parameter space, result in predicted EnHLs between 6.68 and 10.96 ms, based on the results of analysis of simulated signals. The fastest locomotor velocity provided the longest duty cycle values and resulted in the longest predicted EnHLs. The slowest velocity conditions were characterized by longer duration activation durations, resulting in slightly longer EnHLs predicted to be associated with these conditions. There was a tendency for data from the steeper incline conditions (20° and 25°) to result in longer durations of myoelectric activity (Hodson-Tole and Wakeling, 2008a), but this is predicted to have little effect on EnHLs. In addition, as activation durations and duty cycles were similar for the three muscles studied, no systematic differences in EnHL were predicted to occur between them.

### EnHLs resulting from assessment of physiological myoelectric signals

**Figure 6:**
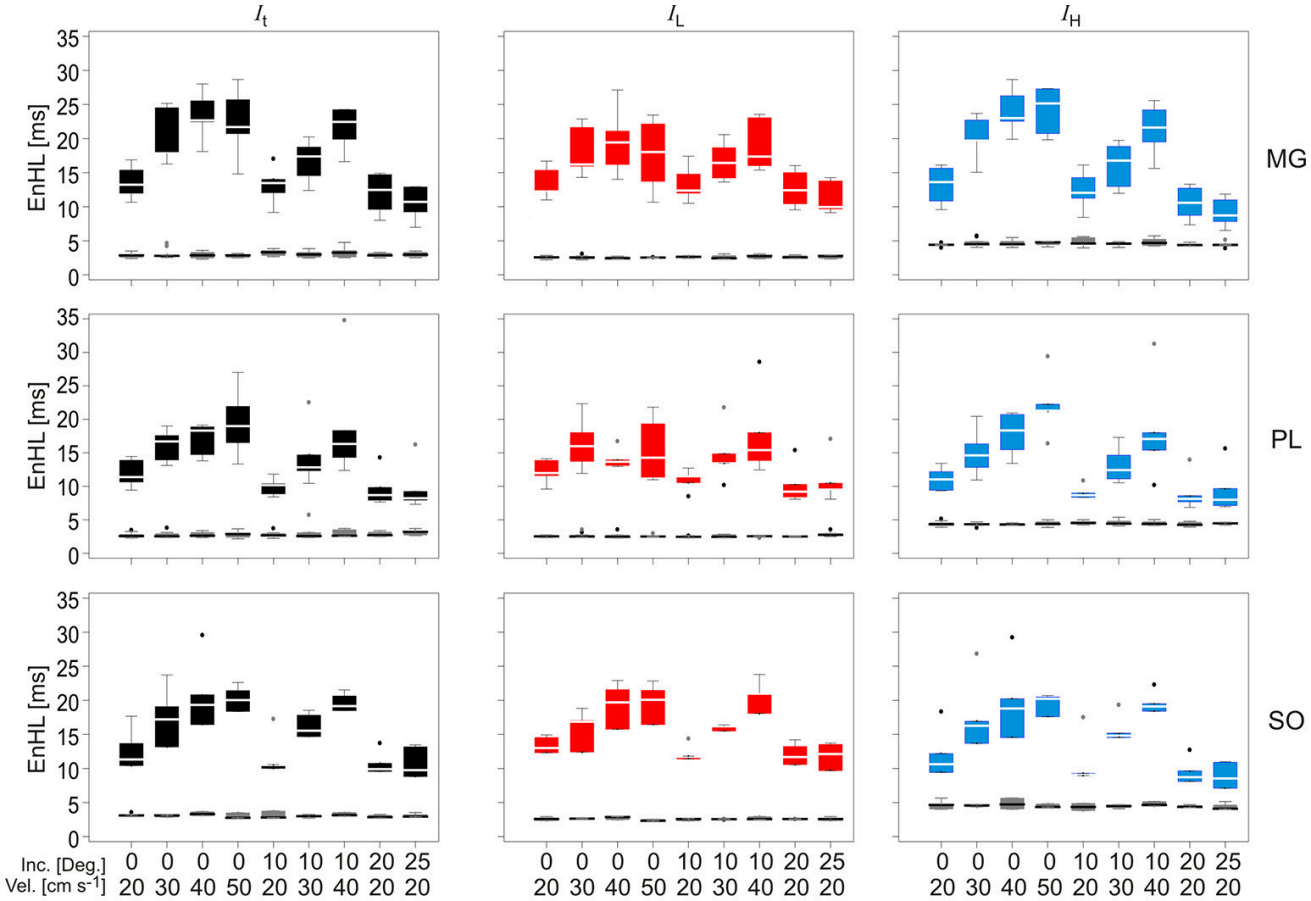
Box and whisker plots of EnHLs [ms] in m. gastrocnemius (Top row), plantaris (Middle row), and soleus (Bottom row) for *I*_*t*_ (Left column, black), *I*_*L*_ (Central column, red), and *I*_*H*_ (Right column, blue). Box and whiskers are also shown for EnHL for phase randomized signals (Gray), where all values were <5 ms. Mid-box marker represents the group median. Upper and lower box edges represent 75 and 25% quantiles, respectively. Upper and lower whiskers represent maximum and minimum values, respectively.

EnHLs calculated from the total (*I*_*t*_), low (*I*_*L*_), and high (*I*_*H*_) frequency intensity components of MG, SO, and PL during each locomotor condition are shown in Figure 6 (also see Supplementary Material). EnHLs were broadly similar across the three muscles studied, with median EnHLs for *I*_*t*_ ranging between 8.22 ms (PL, 25° 20 cm s^−1^) and 22.81 ms (MG, 0° 50 cm s^−1^). These values span a greater range than those predicted from the simulated signals (6.68–10.96 ms, Figure 5). EnHL for *I*_*L*_ ranged between 9.20 ms (PL, 20° 20 cm s^−1^) and 21.02 ms (SOL, 10° 40 cm s^−1^). EnHL for *I*_*H*_ ranged between 8.00 ms (PL, 25° 20 cm s^−1^) and 25.26 ms (MG, 0° 50 cm s^−1^).

Statistical analysis completed for all EnHLs pooled across the different intensity components are shown in Table 1. They reveal highly significant differences (*p* < 0.001) across all factors, except between the three intensity components, although a significant interaction did however occur between intensity component × velocity. Therefore, to investigate differences in EnHL between locomotor conditions (i.e., velocity) within the intensity components analyzed, statistical analysis of factors affecting EnHL were completed separately within each component. A significant effect of muscle was found on EnHL recorded in each signal intensity component (*p* ≤ 0.001 all cases, Table 2), with the shortest EnHL occurring in PL in each case (Table 2). Velocity also had a significant effect on EnHL in each signal intensity component (*p* < 0.001 all cases), with the longest/shortest EnHLs occurring at fastest/slowest velocities in each case (Table 2). This supports the strong velocity effect on EnHL apparent across all three muscles, within each of the intensity envelopes assessed, seen in Figure 6. Incline had a significant effect on EnHL in *I*_*t*_ and *I*_*H*_ (*p* < 0.001, both cases), where the shortest EnHLs occurred during the level running condition (0° slope, Table 2). In this test, no significant interactions between factors were found between muscle, velocity, or incline.

**Table 1:** Group descriptive statistics (median [interquartile range]) for EnHL (ms) calculated from physiological myoelectric signals and results of linear mixed model statistical analysis completed on entire data set.

**Muscle**	EnHL (ms)	*F*_(2,337.31)_ *P*	
MG	16.03 [8.72]	30.10	
PL	13.55 [6.74]	<0.001	
SO	15.64 [7.55]		
**Intensity compon**.	EnHL (ms)	*F*_(2,329.71)_ *P*	*F*_(6,329.71)_ *P*
*I*_*t*_	11.09 [4.21]	1.46	2.54
*I*_*L*_	17.34 [4.45]	0.23	0.02
*I*_*H*_	19.33 [5.41]		
**Velocity** (cm s^−1^)	EnHL (ms)	*F*_(3,329.80)_ *P*	
20	11.39 [4.31]	125.02	
30	16.60 [4.60]	<0.001	
40	19.51 [6.24]		
50	20.73 [4.42]		
**Incline** (Degs.)	EnHL (ms)	*F*_(3,330.15)_ *P*	
0	17.23 [7.32]	14.53	
10	15.18 [6.68]	<0.001	
20	10.43 [4.86]		
25	9.69 [4.14]		

*A significant interaction occurred between intensity component and velocity, so results for this interaction are shown across merged rows (i.e., column furthest right). Statistical tests were completed on Log transformed EnHL, but the original ms values are shown in the table*.

**Table 2:** Group descriptive statistics (median [interquartile range]) for EnHL (ms) calculated from physiological myoelectric signals and results of linear mixed model statistical analysis completed within each intensity component.

	**Total intensity** ******I***_*****t*****_***		**Low frequency intensity** ******I***_*****L*****_***		**High frequency intensity** ******I***_*****H*****_***	
**Muscle**	EnHL (ms)	*F*_(2_, _111.74)_ *P*	EnHL (ms)	*F*_(2_, _111.73)_ *P*	EnHL (ms)	*F*_(2_, _111.40)_ *P*
MG	16.76 [9.53]	10.54	15.37 [6.07]	12.44	16.13 [10.79]	7.65
PL	13.89 [7.00]	<0.001	13.55 [5.32]	<0.001	13.13 [7.78]	0.001
SO	16.82 [8.38]		15.62 [6.92]		14.99 [9.47]	
**Velocity** (cm s^−1^)	EnHL (ms)	*F*_(3_, _103.59)_ *P*	EnHL (ms)	*F*_(3_, _103.24)_ *P*	EnHL (ms)	*F*_(3_, _104.14)_ *P*
20	11.09 [4.21]	39.41	12.17 [3.84]	28.36	10.12 [3.86]	56.31
30	17.34 [4.45]	<0.001	16.21 [4.21]	<0.001	16.34 [5.89]	<0.001
40	19.33 [5.41]		18.07 [5.79]		19.89 [6.59]	
50	21.07 [4.75]		18.81 [7.87]		21.24 [5.02]	
**Incline** (Degs.)	EnHL (ms)	*F*_(3_, _104.03)_ *P*	EnHL (ms)	*F*_(3_, _103.45)_ *P*	EnHL (ms)	*F*_(3_, _104.41)_ *P*
0	17.83 [7.81]	5.39	16.09 [6.11]	2.09	18.32 [7.42]	8.75
10	15.11 [6.61]	0.002	15.29 [5.61]	0.107	14.99 [7.84]	<0.001
20	9.90 [5.13]		11.67 [4.66]		8.74 [4.61]	
25	9.76 [4.71]		10.46 [4.23]		8.72 [3.88]	

*P-values were considered significant when p ≤ 0.017 (i.e., including a Bonferonni correction: 0.05/3). Statistical tests were completed on Log transformed EnHL, but the original ms values are shown in the table. There were no significant interactions between factors*.

**Figure 7:**
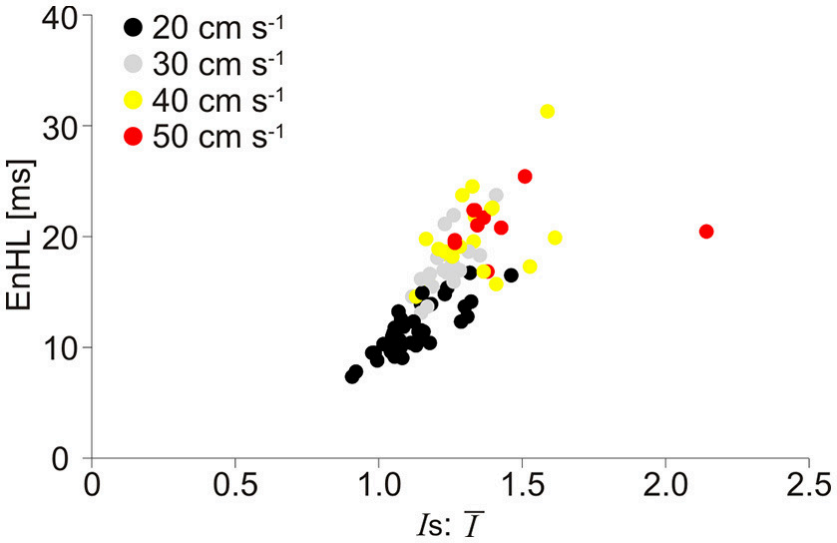
Scatter plot of EnHL as a function of *Is*:Ī from the physiological myoelectric data recorded at different locomotor velocities. *Is*:Ī are taken from each of the three signal components assessed, and each point represents median values for the condition from one participant. Data are pooled across muscle, incline, and intensity component.

The relationship between EnHLs and *Is*:Ī for each analyzed signal component is shown in Figure 7. Lower ratio values occur in the slower velocity running conditions, while the fastest locomotor velocities are associated with larger values. As seen in the simulated data (Figure 4), lower ratios are associated with shorter EnHLs. There is a significant association between the two variables (*p* < 0.001), however the predictive value is low (*r*^2^ = 0.52).

## Discussion

The overarching aims of this study were to: (i) assess myoelectric signal features that influence measures of persistence in signal structure; and (ii) evaluate the resulting predictions of persistence using previously reported physiological data. The outcome measure of persistence in signal structure chosen to be evaluated was EnHL (Zandiyeh and Von Tscharner, 2013; Enders et al., 2015). The combined evaluation of theoretical and physiological data was designed to enable identification of myoelectric signal characteristics that influenced EnHL, evaluate how EnHLs predicted from simulated data compared to results from assessment of physiological signals and assess whether the EnHL metric may reflect neuromuscular responses to locomotor task demand.

From the parameters assessed within the theoretical portion of this work, activation duration and duty cycle were the factors that most affected EnHL. Both these features of muscle activation varied across the mechanical conditions studied in the reports of humans cycling (Blake and Wakeling, 2015) and rodents running (Hodson-Tole and Wakeling, 2007, 2008b, 2010). Using the reported activation durations and duty cycles resulted in EnHLs between 6.68 and 40.00 ms being predicted to occur in physiological data. Reanalysis of myoelectric data recorded from running rats yielded EnHLs well within these ranges. Importantly, EnHL values were significantly different between the original and phase randomized signals, both simulated and physiological, indicating that phase related structure (i.e., the position of each of the data points in time) within them underpinned the EnHLs found. Therefore, changes in EnHL reflect changes in the underlying structure of the recorded myoelectric signals, likely related to motor unit recruitment and firing rates.

Longer EnHLs are suggested to be indicative of signal structure persisting over longer time scales (Zandiyeh and Von Tscharner, 2013). Analysis of the phase randomized signals revealed a strong negative relationship between mean frequency of the EMG intensity and EnHL, such that lower mean frequency was associated with longer EnHL. Whilst these values occur over relatively small ranges (71.8–124.7 Hz; 5.1–8.0 ms), they do evidence that longer EnHL can reflect greater persistence in signal structure given that lower frequencies are associated with longer oscillation periods. When comparing the phase randomized and original signals, there was no difference in their respective mean frequency. However, no relationship was found between mean frequency and EnHL in the original signals, and they were found to provide significantly longer EnHLs. Other signal characteristics, not retained during phase randomization, therefore additionally contribute to the signal structure and the EnHL. From the assessment of the simulated signals, the ratio of the standard deviation and mean signal intensity is a contributing factor with lower ratio values associated with shorter EnHLs (Figures 3, 4), suggesting that the relationship between signal size and variability is important.

Entropy based analyses of myoelectric data are becoming increasingly common in the literature. It is clear that these approaches provide new insight into physiological phenomena such as post-stroke spasticity (Yeh et al., 2016) and motor unit remodeling due to aging (Siddiqi et al., 2015). The only previous report of EnHL from physiological myoelectric data however, were reported for human lower limb muscles and recorded during cycling (Enders et al., 2015). The focus of the work was however on EnHL as a measure of changes in multi-muscle co-ordination, not features of activation within individual muscles as studied here. Nonetheless, Enders and colleagues reported EnHLs of 9 and 16 ms for cycling at 150 and 300 W (90 rpm), respectively. These values are within the range of EnHLs predicted from the theoretical evaluation here (Figure 5). Enders and colleagues did not provide details of myoelectric activation duration or duty cycle of individual muscles in their work, so direct comparison between results is not possible. However, Blake and Wakeling included cycling at 80 and 100 rpm with loads of 200 and 300 W (Blake and Wakeling, 2015). Taking the activation duration and duty cycle values from their work leads to EnHL values of 25.89–23.45 and 13.77–17.88 ms being predicted for 100 and 300 W at 80 and 100 rpm, respectively. These are close to those reported by Enders et al. (2015). Enders et al. however predicted longer EnHLs for the higher load condition whereas the higher load is predicted to lead to shorter EnHLs based on the simulation results here (Figure 5). So while the values are similar the direction of change is opposite.

Given the different system levels assessed by the two studies (here individual muscles; Enders et al. multi-muscle co-ordination) the fact that the EnHLs are so close could be seen as surprising. Such similarity may indicate common features of cyclical patterns of activation are present at both the individual and multi-muscle level, and that these features influence the EnHL metric. Interplay between activation duration and duty cycle may be considered persistent and important myoelectric features, spanning individual, and multi-muscle co-ordination, that are quantified by EnHL. The discrepancy in the direction of change of the EnHL values between the mechanical power outputs studied could reflect two factors: (i) differences in features of individual muscle activation and multi-muscle co-ordination are important in determining EnHL, indicating that the metric may represent quantification of different neurophysiological factors depending on the system level analyzed; and (ii) additional characteristics within physiological signals that influence EnHL were not accounted for within our simulations. Assessing the co-ordination patterns across multiple muscles is unfortunately beyond the scope of the simulation approach used here, leaving an opportunity for further work. Our reanalysis of previously recorded myoelectric data has however enabled further assessment of differences between simulated and physiological EnHLs and will be discussed further below.

EnHLs from simulated and each of the physiological signal components were similar. In the physiological data, faster locomotor velocities were associated with longer activation duty cycles (Figure 5) and significantly longer EnHLs in each intensity component (Figure 6, Table 2). However, it is clear that the predicted direction of change in EnHL is again opposite between simulated and physiological results. Based on both activation duration and duty cycle of faster velocity conditions, the simulated signals suggest that shorter EnHLs should occur (Figure 5). Characteristics within physiological signals that influence EnHL must therefore not have been accounted for within our simulations.

The root of the discrepancy between simulated and physiological signals appears to relate to the ratio of standard deviation and mean signal intensity (*Is*:Ī). The association between EnHL and *Is*:Ī is similar in both simulated (Figure 4) and physiological data (Figure 7) with lower ratios associated with shorter EnHLs in both cases. The degree of signal variability (*Is*_*t*_) in relation to mean signal magnitude (Ī_*t*_) is therefore a key determinant of EnHL in both data sets. However, in the physiological data slower locomotor velocities were associated with lower ratio of standard deviation to mean signal intensity and shorter myoelectric duty cycles (Figure 5). In simulated data shorter duty cycles were associated with greater ratio of standard deviation to mean signal intensity (Figure 3). Therefore, while the simulation approach has indicated this ratio as a determinant of EnHL it has failed to appropriately predict how it is influenced by myoelectric duty cycle. This factor explains the contradiction between the direction of change in EnHL seen in simulated and physiological results presented. Why the simulated signals did not reflect changes in the ratio of standard deviation and mean signal intensity in the physiological data is unclear. One point to consider is that simulated signals were surface myoelectric interference patterns generated using an abstracted model representing a simplified human muscle (Section Skeletal Muscle Model Properties). In contrast the physiological signals were intramuscular recordings from rat muscle. Discrepancies may therefore reflect differences between intramuscular vs. surface signal properties or differences in myoelectric signal properties between species (human vs. rat). A second point to consider is that the quiet phases of the simulated activation cycles would have provided very repeatable patterns of data points. This would lead to lower SampEn and longer EnHL at shorter duty cycles, which is observed in the results (Figure 3). So it is likely this is a significantly contributing factor. Adding a white noise component to simulated signals, did not however affect these results, with a line of unity fitting a plot of EnHLs from “clean” and noise contaminated signals (data not shown, equation for line: −1.65 × 10^−14^+1.0*x*, *N* = 1,440 = 16% of signals tested). This is likely to reflect that the added noise was removed by the signal processing completed prior to EnHL calculation. In physiological signals, information may be contained in the myoelectric signal during periods of quiescence and not be removed during processing. Such signal content could reflect cross-talk from other, nearby muscles or may reflect low levels of voluntary activity or activity related to maintenance of muscle tone. The signals analyzed here were recorded using fine-wire intramuscular electrodes, which are considered to be less susceptible to cross-talk than surface electrodes. Investigating the properties of “quiet” periods of myoelectric signals during cyclical movement patterns may therefore provide additional insight into neuromuscular responses to task complexity, complementing measures from the more traditionally assessed voluntary activation periods.

A third factor that could contribute to differences between the simulated and physiological signals is that only a single motor unit action potential shape was used, whereas physiological signals contain signatures from multiple shapes reflecting populations of activated motor units. The reanalysis of the rat running data incorporated assessment of the signal intensity within low and high frequency bands and the total signal intensity. Including high and low frequency bands enabled assessment of EnHL within faster and slower motor unit populations. As motor unit recruitment patterns can be influenced by the mechanical demand of the locomotor task (Wakeling et al., 2006; Hodson-Tole and Wakeling, 2008a), this is an important feature of myoelectric signals not captured within the simulation where a single, orderly recruitment pattern was used.

While significant differences in EnHL did not occur between the high- and low-frequency intensity components assessed, there was a significant interaction between component and locomotor velocity (Table 1). EnHL differed significantly between velocities within each of the intensity components, and differed significantly between inclines in the total (*I*_*t*_) and high frequency (*I*_*H*_) intensity components, but not the low frequency intensity component (*I*_*L*_, Table 2). Different changes in EnHLs across the locomotor conditions studied were also found between the frequency components within individual muscles (Figure 6). For example, increasing velocity at 0° incline led to similar EnHLs in low frequency intensity component but increasing EnHLs in the high frequency intensity component in PL. This suggests that different changes occur in the structure of signals within high and low frequency bands. Interpreting the frequency bands as representing activation of different motor unit populations, leads to the suggestion that faster and slower motor unit populations may respond differently to alterations in locomotor task demand, which is in agreement with previously published work (Wakeling et al., 2006; Hodson-Tole and Wakeling, 2007, 2008a; Wakeling, 2009b).

As EnHL reflects persistence of signal structure, assessment of the components of myoelectric signals from individual muscles could provide insight into time varying fluctuations in neuromuscular drive to different motor unit populations. As such EnHL could have significant value as a novel marker of neuromuscular responses to changes in the complexity and intensity of a given motor task, enabling persistence of neuromuscular drive to different motor unit populations to be assessed. This has implications for understanding principles that alter neuromuscular drive across different motor unit populations in response to altered locomotor task complexity and intensity, and may provide a novel marker of task demand in the face of injury or pathology (e.g., sarcopenia, spasticity). To extend such line of enquiry it would be valuable to quantify the interplay between signal frequency components and future work may therefore benefit from adapting the EnHL approach applied here, for example by combining it with cross-approximate entropy to evaluate the statistical similarity between two time series (Pincus, 1991). In addition, combined analysis of complexity in myoelectric and other physiologically based signals, such as torque, could be considered to further probe features underpinning phenomena such as fatigue, as recent work, using a number of complexity related metrics, has shown interesting variations in knee extensor torque production during maximal and sub-maximal isometric contractions in humans (Pethick et al., 2015, 2016).

## Conclusions

The theoretical analysis completed here predicts that structure in physiological myoelectric signals from individual muscles, represented by EnHL, persists over time periods of up to 40.00 ms. The predicted EnHLs were influenced by the ratio of standard deviation and mean signal intensity, and mainly reflected interplay between myoelectric activation duration and duty cycle, despite the signals having been filtered to remove stride/cycle frequency components. The EnHLs predicted were close to those previously reported for human cycling (Enders et al., 2015) and those resulting from reanalysis of myoelectric signals from treadmill running rats (Figure 6). The features of the myoelectric signals manipulated for the generation of synthetic signals, can therefore be seen to broadly represent characteristics of physiological features that contribute to EnHL. The direction of EnHL change predicted to occur in response to altered activation duration/duty cycle did not however match those found when physiological data were analyzed. This related to how the ratio of standard deviation and mean signal intensity was affected by activation duty cycle in the simulated data. Therefore, additional content of physiological myoelectric signals, for example content of the quiescent periods in the signal or variation across motor unit action potential shapes, may influence the ratio of standard deviation and mean signal intensity and drive the changes in EnHLs found. How myoelectric signal characteristics from individual muscles manifest within multi-muscle co-ordination patterns has yet to be determined. The potential to assess the complexity and intensity of movement tasks and provide insight into motor control strategies is however significant. Future work should therefore assess whether, as predicted here, features of the ratio of standard deviation and mean signal intensity and activation duration and duty cycle significantly influence EnHL and determine the potential additional utility of EnHL for assessment of neuromuscular co-ordination at this higher system level.

## Ethics statement

Ethical approval relating to rodent data presented was provided by the local ethics committee at The Royal Veterinary College, where all procedures were carried out.

## Author contributions

EHT and JW conceived and designed the study; EHT completed data analysis, drafted the manuscript. JW supported data analysis and helped draft the manuscript. Both authors gave final approval for publication.

### Conflict of interest statement

The authors declare that the research was conducted in the absence of any commercial or financial relationships that could be construed as a potential conflict of interest.

## Data Availability

Results from the analysis of simulated and physiological myoelectric signals are available as Supplementary Material.
