# Supplementary material for: Movement Complexity and Neuromechanical Factors Affect the Entropic Half-Life of Myoelectric Signals
Source: Front Physiol. 2017 Sep 19;8:679. doi: 10.3389/fphys.2017.00679 (PMC5610701; doi:10.3389/fphys.2017.00679)
Supplement: Supplementary file 1 [file SupplementaryInformation.PDF]

Description of column headings for results pertaining to simulated EMG signals (both low and higher firing rate variability):

Act_Level -> simulated activation level ranging from 0.2->1 (steps 0.2), relating to 60->300 motor units. Denoted as 2->10 (steps 2)

Model -> six muscle models with randomly generated motor unit distributions were simulated. Denoted A->F

Act_Duration -> simulated duration of activity ranging 150 - 900 ms (steps of 150 ms)

Duty_Cycle -> five simulated duty cycles ranging 0.3->0.9 (steps 0.2) and 1. Denoted 0_3->1_0 respectively.

Firing_Pat -> five simulations of firing statistics were run. Denoted A, C, E, G and I.

Mean_Sig -> mean signal amplitude (mV)

SD_Sig -> standard deviation signal amplitude (mV)

EnHL -> entropic half-Life (ms)

Entropy -> calculated for m=1 and r=0.25

****
****

Description of column headings for results pertaining to physiological EMG signals:

Muscle -> 1 = medial gastrocnemius; 2 = plantaris; 3 = soleus

Subj -> denotes subject ID code

Incline -> gradient of the treadmill 0, 10, 20 or 25 degrees

Velocity -> locomotor velocity of treadmill 20, 30, 40 or 50 cm/s

EntHL_T -> Entropic half life for the total emg signal intensity (ms)

EntHL_L -> Entropic half life for the low frequency emg signal component(ms)

EntHL_H -> Entropic half life for the high frequency emg signal component (ms);
